# Ubiquitination as an Important Host-Immune Response Strategy in Penaeid Shrimp: Inferences From Other Species

**DOI:** 10.3389/fimmu.2021.697397

**Published:** 2021-05-27

**Authors:** Zhaoxue Zhang, Jude Juventus Aweya, Defu Yao, Zhihong Zheng, Ngoc Tuan Tran, Shengkang Li, Yueling Zhang

**Affiliations:** ^1^ Institute of Marine Sciences and Guangdong Provincial Key Laboratory of Marine Biotechnology, Shantou University, Shantou, China; ^2^ STU-UMT Joint Shellfish Research Laboratory, Shantou University, Shantou, China; ^3^ Southern Marine Science and Engineering Guangdong Laboratory, Guangzhou, China

**Keywords:** ubiquitination, penaeid shrimp, pathogens, immune response, posttranslational modification

## Abstract

Shrimp aquaculture is an essential economic venture globally, but the industry faces numerous challenges, especially pathogenic infections. As invertebrates, shrimp rely mainly on their innate immune system for protection. An increasing number of studies have shown that ubiquitination plays a vital role in the innate immune response to microbial pathogens. As an important form of posttranslational modification (PTM), both hosts and pathogens have exploited ubiquitination and the ubiquitin system as an immune response strategy to outwit the other. This short review brings together recent findings on ubiquitination and how this PTM plays a critical role in immune modulation in penaeid shrimps. Key findings inferred from other species would help guide further studies on ubiquitination as an immune response strategy in shrimp-pathogen interactions.

## Introduction

Shrimp aquaculture has become one of the world’s most extensive and most valuable farming practices due to increased shrimp consumption. However, the rapid development of shrimp aquaculture comes with several challenges, especially pathogenic infections, which therefore hamper the development of the shrimp aquaculture industry. Penaeid shrimps are particularly vulnerable to various microorganisms, including viruses, bacteria, fungi, and other parasites (1, 2) due to the nature of their environment. Moreover, as invertebrates, penaeid shrimps mainly rely on their innate immune system for protection against pathogens (3).

The innate immune system of shrimp comprises cellular and humoral immune responses, which work in concert to offer protection against microbial infections (4, 5). The cellular arm of the innate immune response is involved several immune functions such as phagocytosis and apoptosis through immune cells such as hemocytes (6–8). On the other hand, humoral immune responses involve non-specific enzymes or factors in hemolymph, such as phenoloxidase, lectins, antimicrobial peptides (AMPs), etc., which eliminate pathogens by direct killing or inhibit their growth and spread (2, 3, 9–11). In addition to the direct elimination of pathogens through immune factors in shrimp body fluids, shrimp can also identify pathogens through pattern recognition receptors (PRRs) to activate a series of downstream immune signaling pathways that mediate the activation of cellular and humoral immune responses (12, 13). For example, the carbohydrate recognition domain (CTLD) of C-type lectins (CTLs) in *Marsupenaeus japonicus* recognizes bacterial glycans, while the coiled-coil domain (CCD) interacts with the surface receptor, Domeless, to activate JAK/STAT signaling pathway, which then regulates AMPs production to clear bacterial pathogens (14). Similarly, the Toll and immune deficiency (IMD) pathways are essential in shrimp antimicrobial response due to their major role in regulating AMPs expression *via* the NF-κB/Relish signaling pathways (15, 16).

A growing number of studies have shown that PTMs play an essential role in regulating immune responses (17–20). For instance, phosphorylation of Ser349 on tumor necrosis factor receptor-related protein 3 (TRAF3) by the serine-threonine kinase CK1ϵ facilitates the production of antiviral cytokines. Thus, deficiency of CK1ϵ in mice attenuates its ability to effectively produce antiviral factors during viral infection (21). In *M. japonicus*, the phosphorylation of focal adhesion kinase (FAK) promotes the activation of multiple immune signaling pathways, including antiviral response during white spot syndrome virus (WSSV) infection (22). The envelope proteins, gp116 and gp64, of yellow head virus (YHV), possess N-linked glycosylation (23), which accelerates the formation and release of YHV virions into the hemolymph of infected shrimp (*Penaeus monodon*), hence increasing the severity of the disease (24). Similarly, we previously revealed that the glycosylation of *Penaeus vannamei* hemocyanin increases its antibacterial and agglutination activity against *Vibrio alginolyticus* and *Vibrio fluvialis* (25). In mammals, infection of mouse embryo fibroblasts (MEFs) with herpes simplex virus type 1 (HSV-1) and vesicular stomatitis virus (VSV) induces acetylation of the tumor suppressor p53 at Lys379, which is indispensable for the transcriptional activation of p53-dependent genes in response to viral infection and replication (26). Acetylation of interferon regulatory factor 3 (IRF3) at Lys359 by lysine acetyltransferase 8 (KAT8) results in the inhibition of the transcriptional activity of IRF3 in mouse macrophages, hence, decreasing type I interferons (IFN-I) production and antiviral responses (27).

Recent studies have implicated other PTMs such as small ubiquitin-related modifier (SUMO) modification (SUMOylation) and ubiquitination in immune response in both vertebrates and invertebrates (28–31). For instance, in the retinoic acid–inducible gene I (RIG-I)-like receptors (RLRs) signaling pathway in mice, ubiquitination of RIG-I and mitochondrial antiviral signaling protein (MAVS) affects downstream TNF receptor-associated factors (TRAFs) and IFN regulatory factors, thereby stimulating IFN-I production for antiviral immunity (32–34). In zebrafish, tripartite motif proteins (TRIMs) when induced during viral hemorrhagic septicemia virus (VHSV) infection, display E3 ubiquitin ligase activity, which suggest that TRIMs could regulate antiviral response *via* ubiquitination (35). The viral immediate early (IE) proteins of WSSV can be modified by SUMOylation, thus promoting viral gene transcription and replication in *Procambarus clarkii* (36). During *Staphyloccocus aureus* and *Vibrio parahaemolyticus* infection in Chinese mitten crab *Eriocheir sinensis*, the ubiquitin-like protein, neural precursor cells expresses developmental downregulation 8 (NEDD8) is inhibited to prevent its conjugation with Cullin4 (37), which attenuates the E3 ubiquitin ligase activity of Cullin4 (38). For penaeid shrimps, there is limited information on the role of ubiquitination in immune response. This paper reviews recent findings on the significance of ubiquitination during immune response to microbial pathogens and identify areas that could be explored to understand the role played by ubiquitination in penaeid shrimp immune defense.

## Ubiquitination in Host-Pathogen Interactions

**Figure 1:**
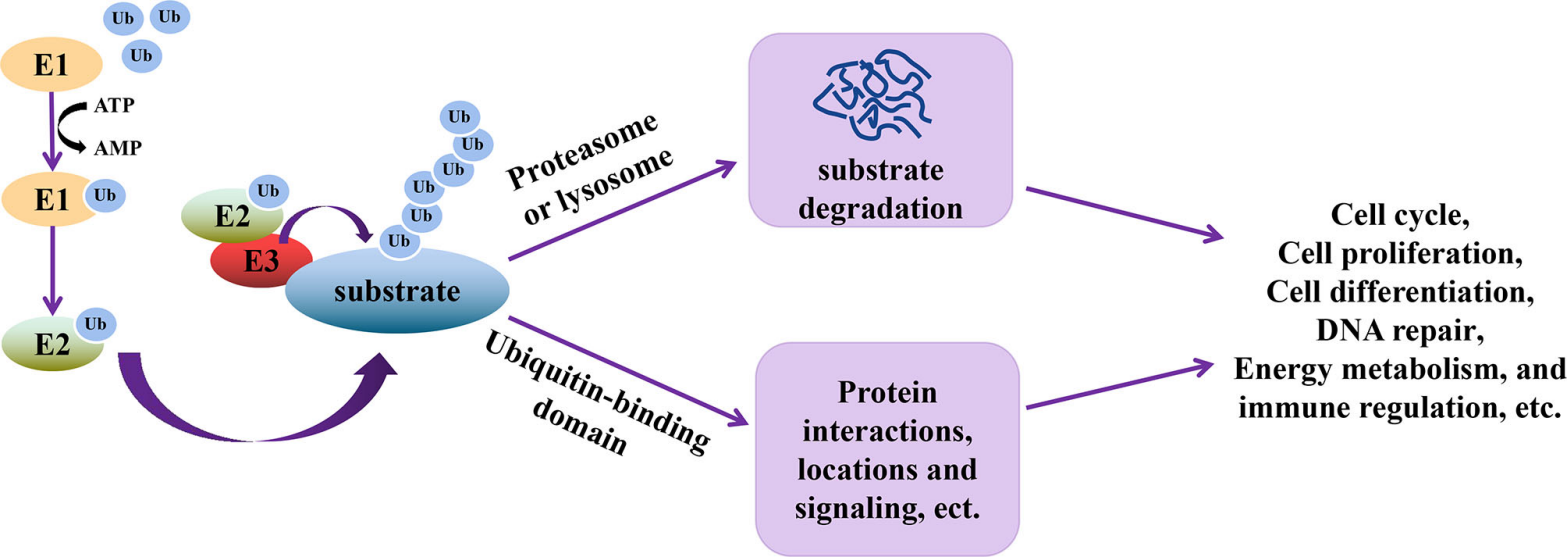
Schematic representation of the functional relevance of ubiquitination. During ubiquitination, E1 is first activated to transfer ubiquitin (Ub) to E2 using ATP. Through the interactions of E2 and E3, E3 and ubiquitinated substrates, Ub is transferred to the substrates, which allows the substrates to be recognized by enzyme complex or organelles. The ubiquitinated substrate can then perform multiple functions, including cell cycle, proliferation, differentiation, DNA repair, energy metabolism, signal transduction and immune regulation, etc.

Ubiquitination is an essential type of PTM, whereby ubiquitin (76 amino acid polypeptides) units are attached to a target protein through a series of three steps catalyzed by ubiquitin-activating enzyme (E1), ubiquitin-conjugating enzyme (E2), and ubiquitin-ligase (E3) (39). In this cascade reaction, E1 activates and transfers ubiquitin to E2 using ATP, followed by the interaction between E2 and E3, allowing the transfer of ubiquitin to the target protein. The seven lysine residues (Lys6, Lys11, Lys27, Lys29, Lys33, Lys48, and Lys63) on ubiquitin or its N-terminal methionine are the residues through which other ubiquitin units bind to increase the number of ubiquitin molecules on the target protein (40). Ubiquitination of target proteins allows these proteins to be recognized by other enzyme complexes or organelles in the cell (40), thereby executing their functions (**Figure 1**), such as cell cycle, proliferation, differentiation, DNA repair, energy metabolism, etc. (41–44).

Besides the primary functions of ubiquitination, recent studies have implicated ubiquitination as an essential regulatory mechanism in immune response (45, 46). For instance, in the orange-spotted grouper *Epinephelus coioides*, the E3 ubiquitin ligase TRIM8 can inhibit the replication of Singapore grouper iridovirus (SGIV) and red-spotted grouper nervous necrosis virus (RGNNV) by enhancing the expression of IR3, IR7 and interferon-related factors (47). During bacterial infection, *Drosophila* E3 ubiquitin ligase LUBEL catalyzes the conjugation of IκB kinase γ (IKKγ) Kenny to form M1-linked linear ubiquitin (M1-Ub) chains, which activates Relish-mediated AMPs gene expression to clear the bacteria (48). In *Scylla paramamosain*, an E3 ubiquitin ligase casitas B-lineage lymphoma (CBL) protein has been found to decrease WSSV proliferation through hemocytes apoptosis (49).

### Ubiquitination and Shrimp Antiviral Response

The key to successful virus infection is the entry of the virus into cells, hence, most viruses use host receptor proteins to enter cells by endocytosis followed by exploiting host nutrients for its replication, proliferation, etc. For example, the WSSV envelope protein, VP24, interacts with polymeric immunoglobulin receptor (pIgR) to enable endocytosis of WSSV into cells, through a pIgR-Calmodulin-Clathrin mediated mechanism in *M. japonicus* (50). Similarly, the WSSV protein kinase 1 interacts with ferritin to block the binding of ferritin to free iron, which facilitates the use of iron by the virus for its proliferation in *M. japonicus* (51). Some viruses also exploit or hijack the immune signaling pathways in shrimp, such as Toll/IMD-nuclear factor-κB (NF-κB), JAK-STAT, and Wnt/β-catenin signaling pathways, for their proliferation (7, 52). For instance, upon WSSV stimulation, NF-κB factor Relish binds to the promoter of the immediate early gene of WSSV, *ie1*, to induce its expression and WSSV replication (7). Toll4 is reported to mediate the production of AMPs through the Toll4-dorsal pathway as an antiviral immune response in *P. vannamei* (16). After JAK/STAT silencing in *P. vannamei*, WSSV copies decreased significantly, indicating that the JAK/STAT signal pathway plays an important role in the antiviral activity of shrimp (53, 54).

In mammals and *Drosophila*, ubiquitination plays a role in mediating innate immune response regulation (46, 55). The E3 ubiquitin ligase RNF125 in mammals, promote K48-linked polyubiquitination and degradation of the mitochondrial adaptor TRIM14 to suppress IFN-I production upon Sendai virus infection (56). Similarly, polyubiquitination of IMD at Lys137 and Lys153 by the E2 ubiquitin-conjugating enzymes (i.e., Effete (Ubc5) and Bendless (Ubc13)-Uev1a complex) and E3 ubiquitin ligase DIAP2 in *Drosophila*, triggers the removal of K63-linked polyubiquitin chains, thereby increasing K48 polyubiquitination to degrade IMD *via* proteasome degradation as part of immune response homeostasis (55). Some emerging evidence suggests the involvement of ubiquitination in mediating antiviral immune response in shrimp. For instance, transcriptome analysis revealed dysregulation in ubiquitin-proteasome pathway genes in shrimp hemocytes (57), while ubiquitination in gills (58) was found to alter WSSV infection of *P. vannamei*, which suggest the modulation involvement of the ubiquitin system in shrimp during WSSV infection. In *F. chinensis*, the ubiquitin-conjugating enzyme FcUbc, was shown to ubiquitinate the really interesting new gene (RING) domains (WRDs) of WSSV viral proteins WSSV277 and WSSV304, to inhibit viral replication and reduces shrimp mortality upon WSSV infection (59). Thus, ubiquitination can play a direct antiviral role in shrimp.

**Figure 2:**
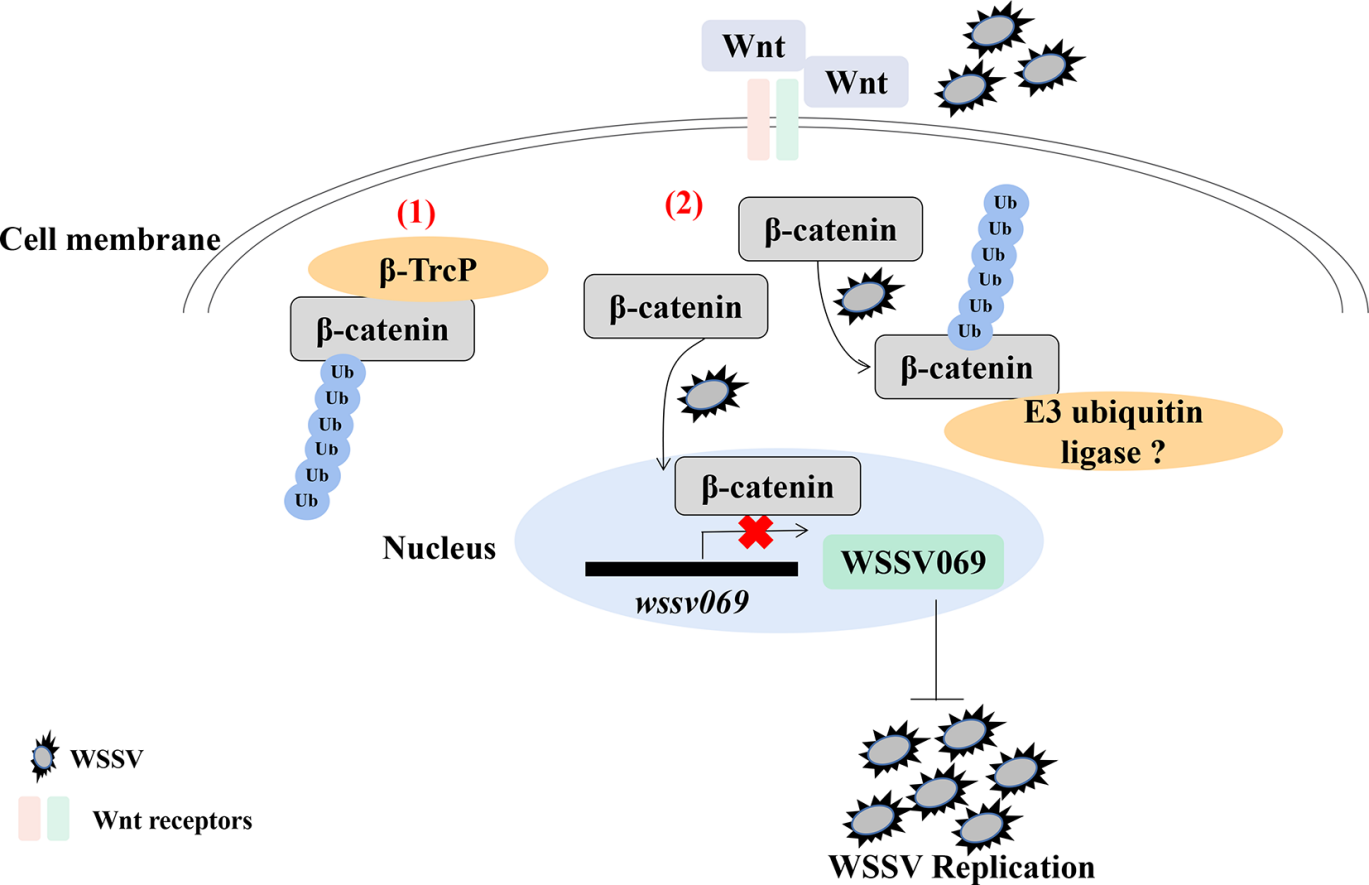
Ubiquitination of β-catenin is important for penaeid shrimp antiviral immune response. β-catenin is a key regulator in the Wnt/β-catenin signaling pathway. (1) In mammals, β-catenin in cytoplasm could be ubiquitinated by an E3 ubiquitin ligase β-TrcP for degradation (62, 63). (2) In the cytoplasm of shrimp, β-catenin is ubiquitinated by WSSV infection, which also promotes the translocation of β-catenin into nucleus to inhibit the expression of virus immediate early gene *wssv069*. β-catenin can also interact with wssv069 (61). Thus, β-catenin plays a positive role through ubiquitination to inhibit WSSV replication during infection. However, which protein could ubiquitinate β-catenin in penaeid shrimp has not been found.

Analysis of the hepatopancreas transcriptome of WSSV infected shrimp (*P. vannamei*) revealed decreased expression of Wnt signaling pathway genes (60). Indeed, ubiquitination is reported to regulate the Wnt/β-catenin pathway in shrimp, as β-catenin undergoes ubiquitination after treatment with the proteasome inhibitor MG132 followed by WSSV infection (61). Given that Pvβ-catenin positively regulates AMPs production (52), interacts with WSSV069, and attenuates the expression of viral genes (61), it indicates that ubiquitination plays a positive antiviral role in shrimp through β-catenin in the Wnt/β-catenin pathway, especially during early viral replication (**Figure 2**). Besides, ubiquitination is involved in antiviral immune response through other proteins by indirectly affecting immune signaling pathway. For instance, the E3 ubiquitin ligase tripartite motif 9 protein (PvTRIM9) interacts with the NF-κB pathway inhibitor beta-transducin repeat-containing protein (Pvβ-TrCP) in *P. vannamei*, hence knockdown of PvTRIM9 followed by WSSV infection increases PvRelish expression and AMPs production, coupled with a decrease in viral copy number (64). This indicates that WSSV inhibits the NF-κB pathway and AMPs production by annexing the E3 ubiquitin ligase PvTRIM9 in shrimp (64).

### Exploitation of Ubiquitination by Viruses to Outwit Penaeid Shrimp Immune Response

Viruses invade their host through interactions between host and viral proteins, especially *via* the use of viral envelope proteins for recognition and entry (7, 65), recruiting cellular proteins in order to invade the cytoplasm (7, 66), etc. Furthermore, viruses can manipulate and exploit the host ubiquitin system to elude host immune response (67). For instance, the immediate-early 1 protein (IE1) of congenital human cytomegalovirus (HCMV) promotes the ubiquitination and degradation of Hairy and Enhancer of Split 1 (Hes1), an essential downstream effector of Notch signaling (68). By this, IE1 acts as an E3 ubiquitin ligase to dysregulate Notch signaling, thereby resulting in aberrant differentiation of in neural progenitor cells (NPCs) (68, 69). Similarly, proteins that contain the RING domain are reported to act as an E3 ubiquitin ligase, such as TRIM proteins (70, 71). In *E. coioides*, the TRIM32 protein, which has a deletion of the RING domain, cannot positively regulate the expression of IFN-stimulated genes, hence attenuating antiviral response to SGIV or RGNNV infection, an indication of the importance of the RING domain in host antiviral response (72). Recent studies show that some WSSV proteins, such as WSSV199, WSSV222, WSSV249, and WSSV403, contain the RING domain; hence, they modulate the ubiquitin system of shrimp to benefit (73). For instance, in the presence of shrimp ubiquitin-conjugating enzyme UbcH6, WSSV222 acts as E3 ubiquitin ligase to mediate ubiquitination and degradation of shrimp tumor suppressor-like protein (TSL), which aids viral replication (74). Furthermore, knockdown of WSSV222 by small interfering RNA (siRNA) reduces the severity of WSSV infection by delaying WSSV replication in shrimp (75). The WSSV222 viral protein, therefore, promotes WSSV replication by annexing the ubiquitin system in shrimp during WSSV infection. Similarly, the viral protein, WSSV403, is reported to exert its E3 ubiquitin ligase activity with shrimp ubiquitin-conjugating enzyme E2 (76). WSSV403 can interact with protein phosphatase (PPs) in shrimp, which suggests that WSSV403 uses its E3 ubiquitin ligase activity to regulate WSSV latent infection (76). However, whether PPs are the ubiquitinated substrate of WSSV403 under WSSV infection has not been reported. Details on this would identify new strategies or potential targets for preventing and treating WSSV infection in shrimp.

### Ubiquitination as an Immune Response to Other Microorganisms or Environmental Cues in Penaeid Shrimp

During host-pathogen interactions, the host can regulate its ubiquitin system to eliminate the pathogens (30). For example, a 74 amino acid residue containing ubiquitin obtained from gills of Pacific oyster *Crassostrea giga* shows strong antimicrobial activity against Gram-positive (e.g., *Streptococcus iniae*) and Gram-negative bacteria (e.g., *Vibrio parahemolyticus*) (77). Host cells can eliminate pathogens through ubiquitination and degradation mediated by proteasome, phagosome, and autophagosome (30, 78). In MEFs, an E3 ubiquitin ligase, neuronal precursor cell expressed, developmentally downregulated 4 (NEDD4), promotes autophagy to clear bacteria *via* ubiquitination of BECN1 (79). During infection with fungi or Gram-positive bacteria, *Drosophila* Spätzle activates the Toll receptor, resulting in the ubiquitination and proteasome degradation of the Toll receptor adaptor protein Cactus, thereby enhancing AMPs production to clear the pathogens (31). Apart from the modulation of the ubiquitin system by the host during an immune response, some pathogens also exploit or interfere with the host ubiquitin system to replicate and escape immune surveillance (80). For example, the NleL ligase (Non-Lee encoded effector ligase), a virulent protein of enterohemorrhagic *Escherichia coli* (EHEC), acts as an E3 ubiquitin ligase that ubiquitinates the Lys68 of human c-Jun NH2-terminal kinases (JNK), which results in the dephosphorylation and deactivation of JNK to enhance EHEC infection (81).

**Figure 3:**
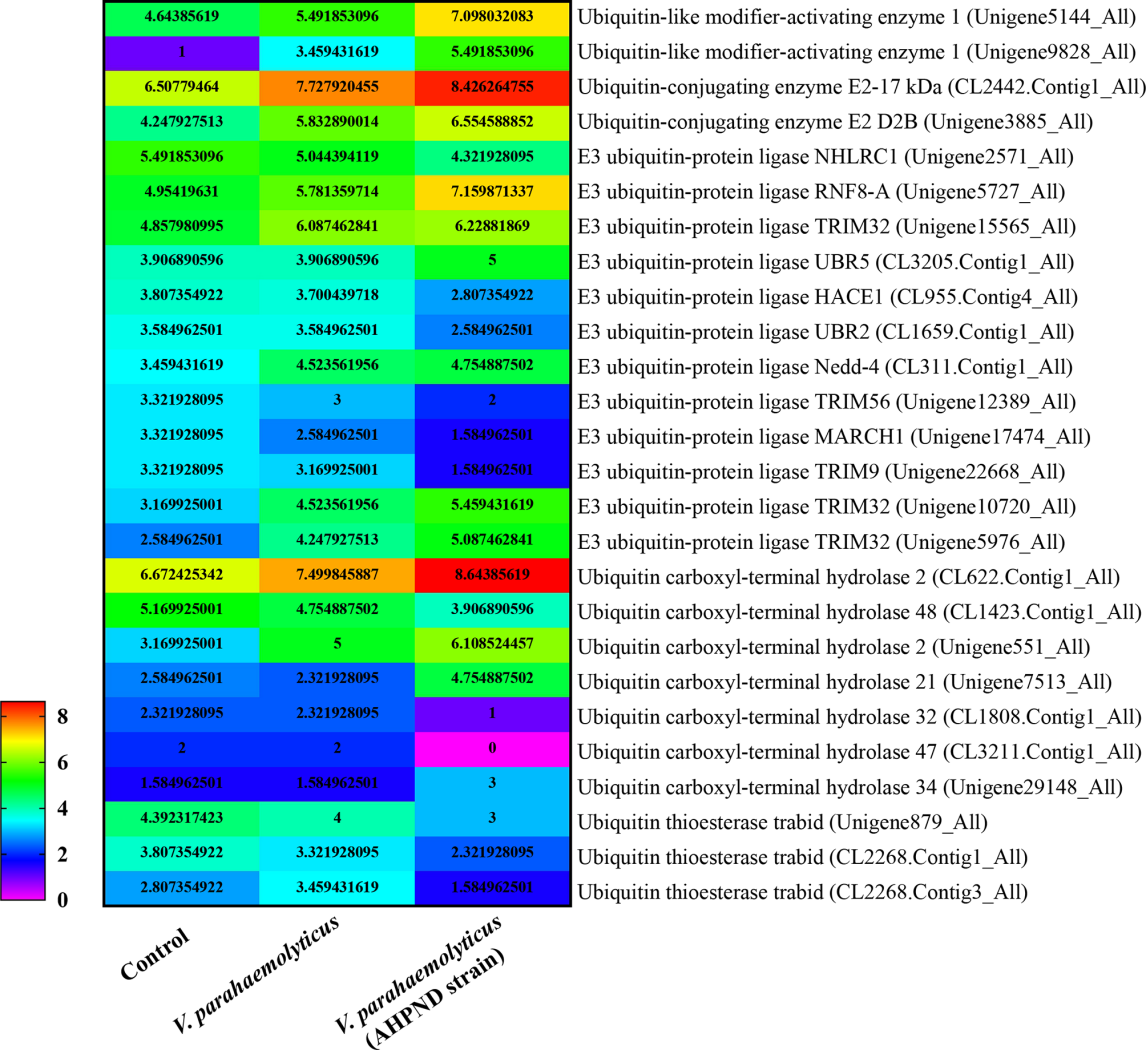
|eat map showing changes in the expression pattern of ubiquitin-related genes in penaeid shrimp (*Penaeus vannamei*) hemocytes infected with *V. parahaemolyticus* and *V. parahemolyticus* (AHPND strain). The numbers represent Log2 fold change. Hemocytes samples were pulled from 30 individual shrimps (n=30). Data used for the figure was culled from the transcriptome data of (83).

**Figure 4:**
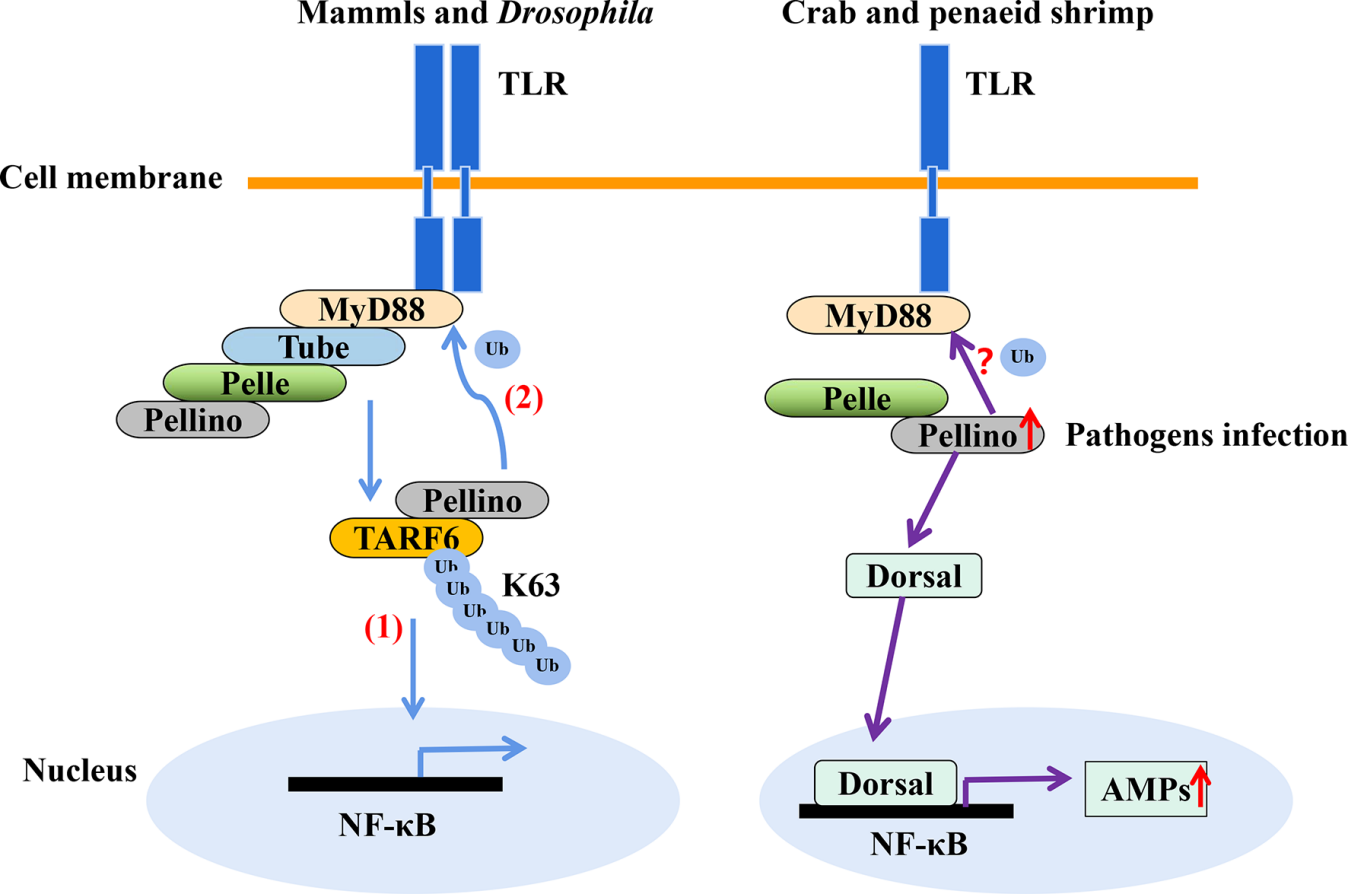
The E3 ubiquitin ligase Pellino plays a positive role in shrimp antibacteria response. Pellino acts as a conserved scaffold protein and E3 ubiquitin ligase in the TLR signaling pathway. (1) Pellino promotes the K63-linked ubiquitination of TARF6 (88). (2) In addition, Pellino can ubiquitinate MyD88 for degradation to negatively regulate TLR signaling and maintain innate immune homeostasis (89). Pellino can also interact with Pelle in shrimp. The expression of Pellino is up-regulated upon pathogens stimulation, and to increase the activity of Dorsal, thus enhancing AMPs expression with *V. parahaemolyticus* challenge (85), in which Dorsal is also involved in defense against gram-positive bacterial in *S. paramamosain* (90). However, the ubiquitinated substrates of Pellino in shrimp (such as whether MyD88 is ubiquitinated) is currently unknown.

In shrimp, few studies have explored ubiquitination as an immune response to invasion by pathogens other than viruses. Nonetheless, transcriptomic analysis of hemocytes from lipopolysaccharide (LPS) treated *P. vannamei* revealed the upregulation of some ubiquitin-proteasome pathway genes including ubiquitin, ubiquitin-conjugating enzyme E2C, ubiquitin-conjugating enzyme H1 and ubiquitin-conjugating enzyme H5b (82). Similarly, ubiquitin-related genes, such as ubiquitin, ubiquitin-activating enzyme E1, ubiquitin-conjugating enzyme E2, various E3 ubiquitin ligases and deubiquitinating enzymes, were significantly changed in the transcriptome of hemocytes from *P. vannamei* infected with acute hepatopancreas necrosis disease (AHPND) *V. parahemolyticus* (**Figure 3**) (83). Significant changes in the expression of ubiquitin-mediated pathway genes have also been found in the transcriptomic study of hemocytes from *V. parahaemolyticus* infected mud crab *S. paramamosain* (84). All these studies indicate a strong association between the ubiquitin system and bacterial infection in crustaceans. Although there is currently no sufficient evidence in penaeid shrimps relating the use of the ubiquitin system by host to clear invading bacteria or it being hijacked by pathogens to invade immune clearance, a number of studies including *in vivo* knockdown of the E3 ubiquitin ligase Pellino in *P. vannamei*, have shown decreased AMPs production and increase shrimp mortality upon *V. parahaemolyticus* challenge (85). This suggests that PvPellino plays a positive role in the antibacterial response in shrimp. However, the specific role of Pellino in shrimp antibacterial response and its ubiquitinated substrate has not been reported. In any case, Pellino proteins are involved in the TLR signaling pathway, acting as conserved scaffold proteins and also function as an E3 ubiquitin ligase (86, 87). In mouse macrophages, Pellino is induced by LPS stimulation, thus promoting the ubiquitination of TANK-binding kinase 1 (TBK1) and TRAF6 to regulate the TLR signaling pathway (88). Similarly, Pellino can also interact with and ubiquitinate MyD88 to maintain innate immune homeostasis (89). All these pieces of evidence can be used as references to explore the ubiquitinated substrates of Pellino and its response to microorganisms in shrimp (**Figure 4**).

Prokaryotes have acquired a complex system for hijacking host cells in evolution. As much as most bacterial genome components are used for host infections (91), some bacterial proteins can modulate the host’s immune response as E3 ubiquitin ligases. For instance, in mice infected with *Shigella flexneri*, bacterial E3 ubiquitin ligase invasion plasmid Ag H (IpaH) protein IpaH4.5 promotes K48-linked polyubiquitination of TBK1 for degradation, which inhibits IFN production and contributes to bacteria colonization (92). The ubiquitin-like protein NEDD88 in *Drosophila* modifies Cullin family. The effector protein Cif, derived from EHEC, deaminates NEDD8, thus disrupting Cullins’ modification, which leads to accumulation of cell cycle regulatory factor and cell cycle retardation of the host (93, 94). In addition, 5’ expressed sequence tags (ESTs) analysis of *Perkinsus marinus*, a protozoan parasite of the eastern oyster *Crassostrea virginica*, revealed ubiquitin-specific proteases, which are thought to be involved in the degradation of host protein substrates to obtain normal cell function and proliferation necessary for nutrition (95). Ubiquitin components, proteasome, and autophagy pathway are involved in the defense of *C. elegans* against microsporidian *Nematocida parisii* infection (96). Thus far, no bacterial proteins or proteins from other pathogens have been identified as E3 ubiquitin ligase or found to interfere with the ubiquitin system in shrimps to mediate immune response. It, therefore, indicates that few studies have explored ubiquitination as an immune response feature of shrimp to bacteria, fungi, or other parasites infections.

Besides microbes, environmental factors, such as temperature and ammonia nitrogen stress, can directly affect protein modification. For instance, phosphorylation of p38^MAPK^ in gills of *P. vannamei* increased rapidly under acute cold stress (97). Under chronic low-salinity stress, the acetylation of histone H4 changed significantly in the hepatopancreas of *P. vannamei* (98). In *P. monodon*, the expression of ubiquitin-conjugating enzyme E2 increased significantly in muscle but decreased in gut tissue in response to low salinity stress (99). Moreover, ubiquitin-related proteins, oxygen-free radicals, and oxidative stress-related proteins all changed significantly under long-term low salinity stress in the hepatopancreas of *P. vannamei* (100), which suggest that changes in the ubiquitination pathway in shrimp may be related to detoxification and immunity (100). It has also been reported that environmental factors affect the survival and proliferation of microorganisms. For instance, water temperature changes could promote *Vibrio* abundance in aquatic environments (101, 102). Similarly, low salinity levels could decrease the immune response of *P. vannamei* and attenuate their resistance to pathogens (103). As discussed in this short synthesis, microbial invasion could elicit host immune response *via* the ubiquitin system in penaeid shrimp. It is therefore conceivable that aquatic environmental factors such as temperature and salinity could also impact on ubiquitination thereby directly or indirectly affecting microorganisms and their interaction with hosts.

## Summary and Future Perspective

Aquatic pathogens are still a threat to shrimp aquaculture. Ubiquitination regulates many biological processes in cells, including host immune response to pathogens. The synthesis presented in this paper provides a snapshot of ubiquitination as a host-pathogen immune response strategy in shrimp. Most current studies have shown that microbial infection of shrimp could induce ubiquitination through ubiquitin-conjugating enzymes, ubiquitin ligases, or ubiquitinated substrates (64, 85, 104). On the one hand, the ubiquitin system in shrimp could be exploited as a mechanism to resist pathogen invasion or annexed by microbial pathogens for replication and survival. For instance, WSSV viral proteins WSSV277 and WSSV304 are ubiquitinated by FcUbc, thereby inhibiting WSSV replication in Chinese white shrimp *F. chinensis* (59), while ubiquitination of β-catenin in shrimp promotes viral replication (61). Pathogens can also exploit the ubiquitin system for their benefit. Some viral proteins act as E3 ubiquitin ligases to promote viral infection and survival using the host ubiquitin system. The RING proteins of WSSV, including WSSV199, WSSV222, WSSV249, and WSSV403, could serve as E3 ligases to coopt the ubiquitin-proteasome pathway of shrimp for viral replication (74, 76, 104). Furthermore, WSSV249 acts as an E3 ligase through interaction of ubiquitin-conjugating enzyme E2 for viral pathogenesis in *P. vannamei* (105). Besides, the ubiquitination pathway might also be related to detoxification and shrimp immunity with salinity stress (100).

Although an increasing number of studies show that ubiquitination plays a vital role in immune response to pathogens, the detailed mechanisms and key players have not been elucidated in penaeid shrimps. For instance, the E3 ubiquitin ligases of some ubiquitinated proteins have not been identified. Although ubiquitination of Pvβ-catenin is reported to inhibit WSSV replication, the corresponding E3 ubiquitin ligase of Pvβ-catenin in shrimp remains unknown. Furthermore, it is unclear whether penaeid shrimp β-catenin also requires phosphorylation to initiate the ubiquitination process as in mammals. Besides, if phosphorylation of β-catenin does occur in shrimp, whether the regulation of phosphorylation and ubiquitination modification affects the antimicrobial immune function of β-catenin remains to be delineated. Currently, the specific functions of some identified E3 ubiquitin ligases in shrimps remains unknown. For instance, while the E3 ubiquitin ligase activity of PvTRIM9 in *P. vannamei* has been demonstrated, PvTRIM9 involvement in shrimp antiviral and antibacterial response is still unknown. Most reports on the relationship between ubiquitination and immune response in penaeid shrimp have mainly focused on WSSV infection. However, numerous studies have established links between ubiquitination and immune response to several pathogens in other species. For example, depletion of the E3 ubiquitin ligase TRIM62 in mice increases their susceptibility to fungal infection (106). Similarly, TRIM8 (47) and TRIM32 (72) are found to inhibit the replication of DNA (e.g., SGIV) and RNA (e.g., RGNNV) viruses in *E. coioidest*. Treating human peripheral blood mononuclear cells (PBMCs) with the proteasome inhibitor MG132 inhibits the ubiquitin-proteasome system and attenuates the replication of *Plasmodium falciparum* (107).

Given that the ubiquitin-proteasome system is conserved, it is conceivable that ubiquitination also serves as an immune response strategy to fungal and parasite infections in shrimp. Studies could therefore explore the significance and relevance of ubiquitination in penaeid shrimp immune response. A better understanding of the specific role of ubiquitination in host-pathogen interaction as an immune response strategy in penaeid shrimps would provide new avenues for improving shrimp immunity and, therefore, disease prevention. Some key components of the ubiquitin system, such as E1, E2, E3, and the ubiquitinated substrates, could be explored as potential targets to enhance shrimp immunity or treatment (108). Focused studies on the interplay between the ubiquitin system of penaeid shrimp and microbial pathogens would help unravel the molecular mechanism of shrimp immune response and how resistance to pathogens can be improved, which is vital for the development of penaeid shrimp aquaculture industry.

## Author Contributions

JA and YZ conceived the idea. ZXZ and JA performed the literature search, wrote and revised the paper. YZ and JA obtained funding and provided supervision. DY, ZHZ, NT, and SL provided literature input and suggestions. All authors contributed to the article and approved the submitted version.

## Funding

This work was sponsored by the 2020 Li Ka Shing Foundation Cross-Disciplinary Research Grant (No. 2020LKSFG01E), National Natural Science Foundation of China (Nos. 31872596 & 32073008), Key Special Project for Introduced Talents Team of Southern Marine Science and Engineering Guangdong Laboratory (Guangzhou) (No. GML2019ZD0606) and Shantou University Scientific Research Foundation for Talents (No. NTF19005).

## Conflict of Interest

The authors declare that the research was conducted in the absence of any commercial or financial relationships that could be construed as a potential conflict of interest.
